# Complications of Balloon Pulmonary Angioplasty: A Comprehensive Analysis Based on the Latest ESC Consensus Statement

**DOI:** 10.3390/jcm13154313

**Published:** 2024-07-24

**Authors:** Fe J. van Leusden, Diederik P. Staal, Mitch C. J. van Thor, Benno J. M. W. Rensing, Jan-Peter van Kuijk, Berend M. Mulder, Daniël A. F. van den Heuvel, Sanne Boerman, Karin A. Boomars, Joyce Peper, Johannes J. Mager, Marco C. Post

**Affiliations:** 1 Department of Cardiology, St. Antonius Hospital, 3435 CM Nieuwegein, The Netherlands; 2 Department of Radiology, St. Antonius Hospital, 3435 CM Nieuwegein, The Netherlands; 3 Department of Respiratory Medicine, St. Antonius Hospital, 3435 CM Nieuwegein, The Netherlands; 4 Department of Respiratory Medicine, University Medical Center Rotterdam, 3015 GD Rotterdam, The Netherlands; 5 Department of Cardiology, University Medical Center Utrecht, 3584 CX Utrecht, The Netherlands

**Keywords:** percutaneous intervention, pulmonary hypertension, pulmonary embolism

## Abstract

**Background/Objectives:** The literature reports high complication rates in patients with chronic thromboembolic pulmonary hypertension (CTEPH) who undergo balloon pulmonary angioplasty (BPA), especially in patients with poor pulmonary hemodynamics. Here, we describe the complications of BPA based on the new definitions. **Methods:** All patients with CTEPH who completed BPA treatment before 15 September 2023 were selected from the CTEPH database. Peri-procedural complications were collected and classified according to the 2023 consensus paper on BPA treatment. Complications were analyzed in subgroups of patients with pulmonary vascular resistance (PVR), ≤ or >6.6 WU, and mean pulmonary artery pressure (mPAP), ≤ or >45 mmHg, at first BPA. **Results:** In this analysis, 87 patients (63% women; mean age 61.1 ± 14.0 years; 62% on dual PH targeted medical therapy) underwent 426 (mean 4.9 ± 1.6 per patient) BPAs. Only non-severe complications occurred in 14% of BPA treatments and in 47% of the patients; 31% patients had a thoracic complication. The thoracic complications were mild (71%) or moderate (29%). Patients with a PVR > 6.6 WU (*n* = 8) underwent more BPA treatments (6.6 ± 1.5 versus 4.6 ± 1.5, *p* = 0.002), had more complications (88% versus 41% of patients, *p* = 0.020), and had more thoracic complications (17% vs. 7% of BPAs, *p* = 0.013) than patients with PVR ≤ 6.6 WU. Patients with mPAP > 45 mmHg (*n* = 13) also had more BPA treatments (6.5 ± 1.7 versus 4.6 ± 1.4, *p* < 0.001), more complications (77% versus 44% of patients, *p* = 0.027) and more thoracic complications (14% versus 8% of BPAs, *p* = 0.039) than patients with mPAP ≤ 45 mmHg. **Conclusions:** Complications occurred in 14% of BPAs and were mostly mild. Patients with severe pulmonary hemodynamics suffered more (thoracic) complications.

## 1. Introduction

Chronic thromboembolic pulmonary hypertension (CTEPH) is a rare, progressive pathophysiological disorder that is characterized by persistent pulmonary occlusion, resulting in increased pulmonary vascular resistance (PVR), right heart failure and premature death [1,2]. The therapeutic management of CTEPH is multimodal, and consists of pulmonary endarterectomy (PEA), balloon pulmonary angioplasty (BPA) and/or pulmonary hypertension (PH)-targeted medical therapy, depending on the patient and disease characteristics [1]. For patients with proximal obstructive lesions, PEA is potentially curative and the treatment of choice [1,3,4]. For patients who are ineligible for PEA or for patients with residual pulmonary hypertension (PH) after PEA, BPA offers an alternative, percutaneous interventional approach [1,5,6,7,8,9,10]. Originally, BPA was associated with severe reperfusion edema, leading to a 30-day mortality rate of 5.5% [5,11]. Mizoguchi et al. introduced BPA as a multiple-staged procedure in 2012, which improved safety considerably and resulted in a prognostic beneficial method for the treatment of CTEPH patients with distal thromboembolic lesions [5,12].

Peri-procedural complications have been described extensively in the last decade, but until recently no consensus was reached concerning the nomenclature of those complications [1,12,13,14]. In 2023, the first consensus statement on BPA treatment and peri-procedural complications was published by the European Society of Cardiology (ESC) [5]. According to the ESC consensus statement, thoracic complications comprise complications related to the nature of the BPA procedure, the pulmonary arteries, or the underlying pathology, and must be distinguished from non-thoracic complications [5]. Lung injury is the most serious (thoracic) complication of BPA, accounting for more than half of all complication-related deaths [5]. A previous study by Wiedenroth et al. associated thoracic complications with a pulmonary vascular resistance above 6.6 Woods Units (WU) [15]. The RACE trial is a unique randomized controlled trial in which inoperable patients were randomized to BPA treatment or PH-targeted medical treatment with Riociguat [16]. In the RACE trial, (serious) procedure-related adverse events occurred more often in patients with pulmonary artery pressures (mPAP) above 45 mmHg [16]. Also, patients who received treatment with PH-targeted medical therapy prior to BPA suffered fewer complications [16]. Therefore, the ESC guidelines for PH stated that PH-targeted medical therapy should be considered in patients with a poor pulmonary hemodynamic status prior to BPA [1,5].

The aim of this study was to identify and classify all peri-procedural complications of BPA at our center for PH according to the new nomenclature developed by the ESC working group. Additionally, this study aims to describe the complication rate in patients with severe hemodynamics (PVR > 6.6 WU or mPAP > 45 mmHg).

## 2. Materials and Methods

### 2.1. Patient Selection and Data Collection

All consecutive patients who were diagnosed with CTEPH and underwent BPA treatment at our hospital from 2015 until the last fully completed BPA procedure in September 2023 were included in this study. For all patients, demographic information (e.g., age; sex; age at diagnosis; diagnosis, medical history of diabetes mellitus, systemic hypertension, coronary artery disease and/or body mass index above 30 kg/m^2^); clinical parameters (e.g., World Health Organization (WHO) functional class; N-terminal prohormone of Brain Natriuretic Peptide (NTproBNP); and 6-min walking distance (6MWD)), and pulmonary hemodynamics (e.g., mean Right Atrial Pressure (mRAP); mPAP; Pulmonary Artery Wedge Pressure (PAWP); Cardiac Output (CO); and PVR) were collected at the moment of diagnosis, at the first BPA and the last BPA. Information on PH-targeted medical therapy was collected at the time of diagnosis and at the first BPA. All data obtained within six months of the date of diagnosis were considered eligible as baseline characteristics. A right heart catheterization (RHC) was performed during the same session as the first and last BPA. The WHO functional class, NTproBNP and 6MWD were considered valid if they were acquired within 3 months of the first BPA or within 3 months after the last BPA. All data were collected and managed using REDCAP electronic data capture tools hosted at the hospital [17,18]. REDCAP is designed to support secure data capture for research studies [17,18]. This study was approved by the local ethical committee of the hospital (MEC-U; Z18.040).

### 2.2. CTEPH Diagnosis and Therapeutic Management

CTEPH diagnosis and a successive assessment of (multimodal) management were established in an expert multidisciplinary team consisting minimally of two PH physicians (cardiologist and pulmonologist), a PEA surgeon, a BPA interventionist, a PH nurse and a radiologist, according to the European guidelines for PH [1]. In short, CTEPH was diagnosed if the following criteria were met: a minimum duration of 3 months of anticoagulation treatment, pre-capillary pulmonary hypertension as measured through right heart catheterization, signs of chronic pulmonary embolism on pulmonary angiography or computed tomography, and ventilation–perfusion mismatches on a ventilation–perfusion scan [1]. Specific considerations for selecting patients for BPA and/or for PH-targeted medical therapy were described previously in detail [19,20]. In short, patients were eligible for BPA if they were inoperable or were unwilling to undergo PEA, had accessible thromboembolic lesions and did not have severe contraindications (e.g., a right-sided mechanical heart valve). Experienced BPA interventionists performed the BPA procedures, following the same principles published previously by van Thor et al. [19]. To summarize, anticoagulation was maintained using vitamin K antagonists or direct oral anticoagulants. If patients were on vitamin K antagonists, the international normalized ratio was maintained between 2.5 and 3.5. Peri-procedural thromboembolic events were prevented by the administration of intravenous heparin (2500–3000 IU). The femoral vein served as the entry point to access affected pulmonary arteries using a 6F to 9F sheath and a 6F guide wire and catheter (Terumo Corporation, Tokyo, Japan). Jopromide was employed to visualize the affected pulmonary arteries, and upon reaching the target regions with the guide wire, semi-compliant balloons were used for dilation. In the absence of clinical complications, patients were transferred to the pulmonology/cardiology ward and discharged the following day. Subsequent BPA procedures were scheduled as needed, four to six weeks apart. PH-targeted medical therapy was indicated for CTEPH patients with a WHO functional class of at least II at diagnosis. The time between diagnosis and the first BPA was not standardized and depended on the availability of BPA at the time of diagnosis. The COVID-19 pandemic caused prolonged waiting times for BPA.

### 2.3. Peri-Procedural Complications

Peri-procedural complications were retrospectively collected by evaluating all electronic patient files of patients who underwent BPA. Complications were categorized according to the 2023 consensus statement for BPA [5]. The following were defined as thoracic complications: hemoptysis, vascular injury, lung injury, and ‘other’ thoracic complications. Hemoptysis was defined as mild if less than a hand full of blood was recorded, moderate if more than a hand full of blood was recorded and severe if signs of respiratory failure were present. Patients were considered to have vascular injury if wire perforation, pulmonary artery rupture or pulmonary artery wall dissection were observed during the procedure or additional imaging was performed. Vascular injury was divided into vascular injury with or without hemoptysis, and severity was categorized the same way it was for hemoptysis. Pulmonary artery dissection was defined as occlusive or non-occlusive. Lung injury was classified as mild if nasal oxygen was supplied, moderate if non-invasive ventilation was needed, and severe if mechanical ventilation was utilized. Lung injury was classified as immediate if symptoms showed within three hours, or delayed if more than three hours passed after BPA. Other thoracic complications (e.g., lung infection and pulmonary artery thromboembolism) were also recorded.

Non-thoracic complications constituted of contrast allergy, access site complications, complications related to RHC and contrast nephropathy. Contrast allergy was classified as mild if cutaneous signs of allergy were present, moderate if bronchospasms occurred and severe if signs of anaphylactic shock showed. Complications associated with RHC consisted of (temporary) conduction disturbances, (supra-)ventricular arrythmia, and pericardial tamponade. Contrast nephropathy was classified as acute kidney injury or acute kidney disease, for which dialysis was or was not needed. Access site complications were recorded if blood transfusion was required or if false aneurysms were present, or if arteriovenous fistulas developed. The details on the definition and therapeutic management of peri-procedural complications can be found in the ESC consensus statement [5]. Mortality, both in-hospital and during the follow-up period, was noted.

### 2.4. Statistical Analysis

All continuous data are presented as mean ± standard deviation (SD) or as median with interquartile range (IQR), where applicable. All categorical data are presented as the number and percentage of the total number of patients, or as the number and percentage of the total number of BPA treatments. Unpaired *t*-tests were performed to compare the incidence of complications between the subgroups of patients with mPAP > 45 mmHg and PVR > 6.6 WU at the first BPA. The cut-off values for elevated mPAP and PVR were chosen based on previous studies by Wiedenroth et al. [15] and Jais et al. [16]. Patients with unknown mPAP or PVR at the first BPA were excluded from the analysis. The statistical tests were two-tailed and were considered significant if *p* < 0.05.

Statistical analysis was performed using R Core Team (2021). R: A language and environment for statistical computing. R: Foundation for Statistical Computing, Vienna, Austria. URL https://www.R-project.org/. Accessed on 1 June 2023. Version 2023.9.0.463 [21]. The following packages were utilized: “tidyverse” [22] and “gtsummary” [23].

## 3. Results

### 3.1. Study Population and Clinical Characteristics

**Figure 1:**
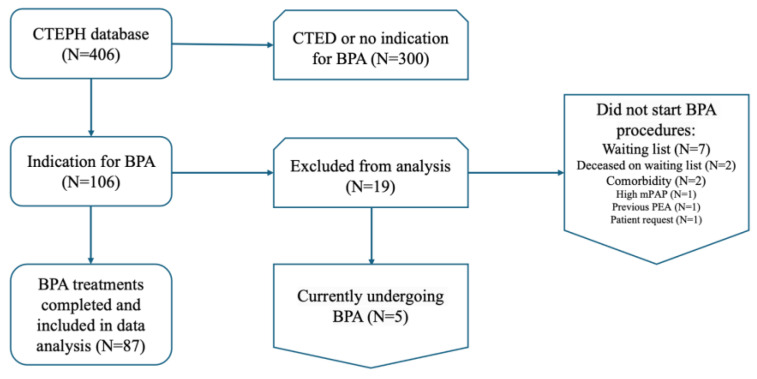
Flowchart of patient inclusion.

In total, 87 patients completed BPA treatments before September 2023 and were included in the present analysis (see Figure 1). Their mean age was 61.1 ± 14.0 years and 63% were female. At baseline, 60% of all patients had a WHO functional class of III/IV; the mean 6MWD was 380 ± 142 m and the median NTproBNP was 344 [108–1788] pg/mL. The baseline mPAP was 38.9 ± 10.0 mmHg and the mean PVR was 6.4 ± 3.6 WU. After diagnosis, 54 (62%) patients received dual PH-targeted therapy. The most prescribed drugs were endothelin receptor antagonists (ERAs) (75%). See Table 1 for all baseline characteristics. Of the 87 included patients, four patients died before they had completed their BPA treatments. One patient died due to acute kidney insufficiency provoked by severe decompensated right heart failure secondary to CTEPH. One patient died due to COVID-19 (*n* = 1), one patient died due to Enterococcus faecalis bacteremia resulting in multi-organ failure (*n* = 1), and for one patient the cause of death was unknown.

### 3.2. Clinical Characteristics during Follow-Up

The median time between diagnosis and the first BPA was 13.8 (IQR 7.4–37.5) months, during which all clinical characteristics improved. At diagnosis, 52 patients (59.8%) had a WHO functional class of III/IV, substantially more than at the first BPA (54.0%) or at the last BPA (17.2%). The 6MWD improved from 380 ± 142 m before the first BPA to 442 ± 126 m after the last BPA. The Log NTproBNP decreased from 5.9 ± 1.8 pg/mL at diagnosis to 5.1 ± 1.2 pg/mL after the last BPA. The mean PAP improved from 38.9 ± 10.0 mmHg at baseline to 29.2 ± 9.6 mmHg after the last BPA. The CO increased from 5.2 ± 1.9 L/min at baseline to 5.7 ± 1.6 L/min at the first BPA, and improved further to 6.0 ± 1.2 L/min after the last BPA. The PVR improved substantially between diagnosis (6.4 ± 3.6 WU), the first BPA (3.7 ± 2.3 WU) and the last BPA (2.7 ± 1.6 WU). See Table 2 for details on the clinical and hemodynamic characteristics.

### 3.3. Peri-Procedural Complications

In total, 426 BPA treatments were performed in 87 patients (4.9 ± 1.6 BPAs per person); of these, 41 (47%) patients suffered at least one complication during the whole treatment period. Most patients had one complication (86%), but some had two (9%), three (2%) or four (2%) complications. During three BPAs, more than one complication was recorded, resulting in a total of 59 complications in 57 BPA treatments, and a complication rate of 14% (59/426 BPA treatments). In total, 19 BPA treatments were performed in patients who underwent prior PEA. During four (21%) treatments, a mild complication occurred; during three procedures, mild hemoptysis was reported and one procedure was complicated by a mild allergic reaction.

Multiple patients had more than one thoracic complication (38 thoracic complications in 26 patients). Hemoptysis (mostly mild) occurred in 24 (6%), and vascular injury in eight (2%) of the BPA treatments, the latter accompanied by hemoptysis in four cases. Vascular injuries included a dissection (*n* = 3), perforation (*n* = 1), the rupturing of a sclerotic wall (*n* = 1), or an unclear mechanism (*n* = 3). Lung injury occurred after four (1%) BPA treatments and was mostly moderate in nature. The signs of lung injury were immediately present in two cases, while the other two cases were delayed. One patient had to be readmitted after the BPA procedure due to bronchial hyperreactivity, which was classified as any other thoracic complication. Another patient suffered from PH syncope during the BPA procedure, for which cardiopulmonary resuscitation had to be performed for less than 30 s.

All signs of contrast allergy were mild. Three complications associated with RHC were supra-ventricular arrhythmia and two complications were temporary conduction disturbances. One patient had signs of temporally acute renal insufficiency, but no dialysis was needed. Six (1%) complications could not be specified according to the new nomenclature for peri-procedural complications: urinary tract infection, bladder retention, collapse due to anxiety, and increased demand for oxygen due to anxiety during the BPA procedure. None of the patients had to be admitted to the intensive care unit and no patients deceased due to complications. All complications are outlined in Table 3.

### 3.4. Peri-Procedural Complications in Patients with mPAP > 45 mmHg and PVR > 6.6 WU

**Figure 2:**
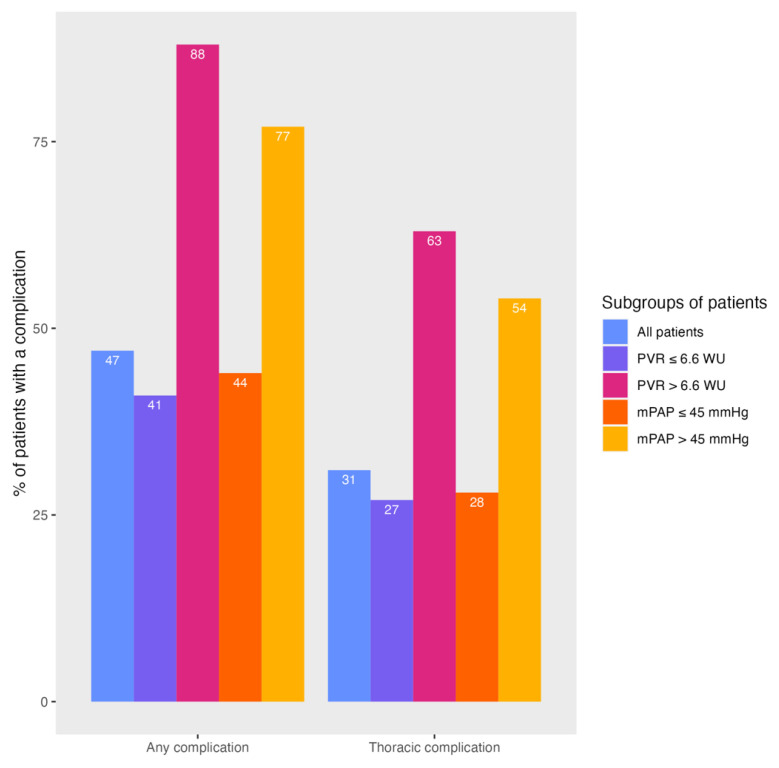
Overview of patients who suffered (thoracic) complications.

**Figure 3:**
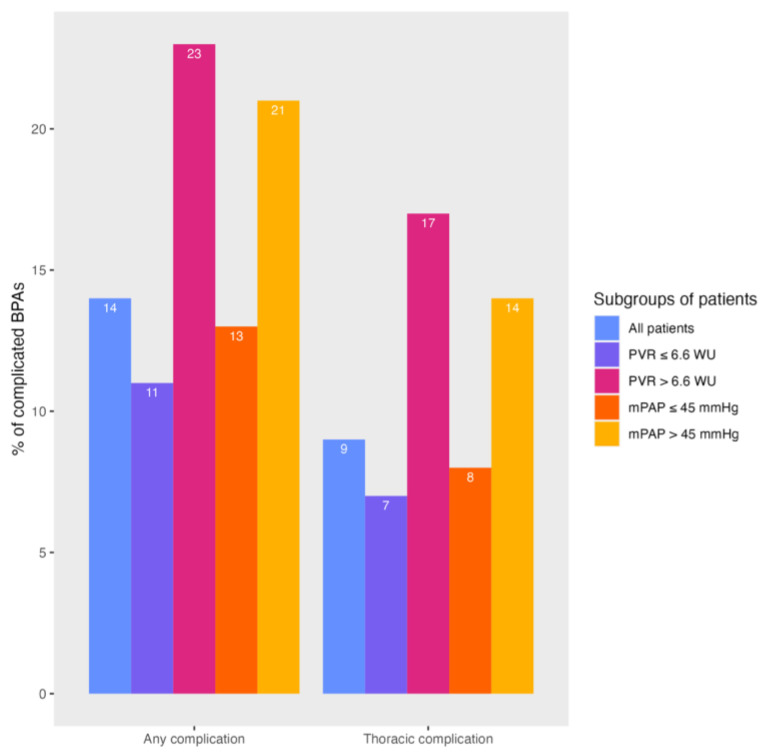
Overview of BPA treatments complicated by (thoracic) complications.

At the first BPA, eight patients had a PVR > 6.6 WU and 13 patients had a mPAP > 45 mmHg. Patients with a high PVR underwent 6.6 ± 1.5 BPA treatments, significantly more than patients with a lower PVR (4.6 ± 1.5, *p* = 0.002). Patients with an increased mPAP also underwent significantly more BPA treatments than patients with a mPAP ≤ 45 mmHg (4.6 ± 1.4 versus 6.5 ± 1.7, *p* < 0.001). Treatments were significantly more often complicated in patients with an increased mPAP or PVR compared to patients with a lower mPAP or PVR (see Figure 2). Also, thoracic complications occurred more often in the presence of a high mPAP (14% versus 8%, *p* = 0.039) or high PVR (17% versus 7%, *p* = 0.013) (see Figure 3). Finally, ‘other’ types of complications also occurred more often in patients with PVR > 6.6 WU (for more details see Table 3). The clinical characteristics of patients with an elevated mPAP and PVR are described in Supplementary Tables S1 and S2. The peri-procedural complications in patients with missing values for mPAP or PVR are described in Supplementary Tables S3 and S4.

## 4. Discussion

This observational study describes peri-procedural complications according to the recent ESC consensus statement on BPA treatments. The main findings from this analysis are that (1) mild peri-procedural complications are frequently observed and that (2) (thoracic) complications, predominantly hemoptysis, are more frequent in patients exhibiting poor pulmonary hemodynamics.

Primarily, the overall prevalence of peri-procedural complications in our study was 14% and 9% for thoracic complications, and none of them were severe. The overall complication rate is comparable to the percentages described in other studies, with a range of 11% to 17% [15,24,25,26]. However, in a recent Japanese study by Ito et al., complications were reported in up to 25% of the BPA treatments [14]. Here, we analyzed mostly inoperable CTEPH patients, in contrast to the Japanese study in which 46% underwent PEA prior to BPA [14]. Previous PEA, as well as the distinctive thromboembolic phenotype observed in Japanese patients, may contribute to the elevated complication rate described by the Japanese investigators compared to the other studies [5,14,25,27,28]. Most studies on peri-procedural (thoracic) complications were reported prior to the publication of the ESC BPA consensus paper, describing the definition of BPA-related complications in detail. Therefore, the comparison of complication rates between different studies should be performed with caution [5]. Given the persistent prevalence of (mild) peri-procedural complications globally, the imperative to categorize peri-procedural complications according to the nomenclature now available must be emphasized for future studies [5].

Wiedenroth et al. associated a PVR > 6.6 WU with an increased likelihood of thoracic complications, particularly pulmonary vascular perforations [15]. To investigate the cut-off value of 6.6 WU further, we analyzed the complications in subgroups of patients with a PVR below and above 6.6 WU. In line with Wiedenroth et al. [15], we found more (thoracic) complications in the presence of a high PVR compared to a lower PVR (17% versus 7%, *p* = 0.013). The thoracic complication rate, however, was mainly driven by peri-procedural hemoptysis rather than pulmonary vascular injuries. In the ESC consensus statement, hemoptysis is reported as an independent thoracic complication, as well as symptom of pulmonary artery injury [5]. While hemoptysis is always the consequence of underlying vascular damage, (additional) imaging is needed to identify vascular injury. In this study, not all patients with hemoptysis received imaging after BPA. Therefore, the prevalence of vascular injuries may be underestimated compared to the prevalence of hemoptysis.

Notably, five out of the six patients (83%) experiencing multiple thoracic complications had a high mPAP and/or high PVR at the first BPA procedure. Among these six patients, four experienced complications of moderate severity. Additionally, 63% of the patients with PVR > 6.6 WU suffered thoracic complications versus 27% of the patients with a PVR ≤ 6.6 WU. In the RACE trial, where patients had a mean baseline PVR of 9.6 WU, 42% of the patients who underwent BPA experienced ≥ 1 treatment-related serious adverse events [16]. These findings suggest that patients with worse pulmonary hemodynamic values are prone to experiencing more, and more serious peri-procedural (thoracic) complications. Accordingly, the imperative to conduct a right heart catheterization at the beginning of BPA treatments and the necessity to monitor individuals with worse pulmonary hemodynamic values closely after BPA must be accentuated [5]. This is especially considering that most peri-procedural complications do not cause significant problems when managed adequately [15].

Furthermore, the reported absence of severe complications and mortality due to BPA-related complications is unique compared to other publications on peri-procedural complications [14,15,26]. In the ancillary follow-up study after the RACE trial, patients who received riociguat prior to BPA had fewer (serious) treatment-related adverse events than patients who underwent BPA without pre-medical treatment [16]. At our center, PH-targeted therapy was prescribed prior to BPA to more than 90% of the patients. Due to the COVID-19 pandemic, the median duration between diagnosis and the first BPA was long (almost 14 months) [16,24]. Between diagnosis and BPA, a notable improvement was observed in most pulmonary hemodynamic and clinical parameters, comparable to the improvements published in an observational study on riociguat treatment prior to BPA [15]. See Table 2 for a detailed overview of the clinical characteristics during follow-up. Interestingly, in 16 patients, the PVR decreased to ≤6.6 WU, and in 10 patients, the mPAP decreased to ≤45 mmHg prior to BPA. Considering that a high mPAP and/or PVR are associated with (thoracic) complications, the administration of PH-targeted medical therapy may have tempered the complication severity and/or influenced the peri-procedural complication rate in our study.

To reduce the peri-procedural complication risk and severity, several factors should be considered. First, prior to BPA, patients at a high risk of (severe) complications should be identified. This requires a thorough pre-procedural work-up to evaluate both disease characteristics. Additionally, optimizing the BPA strategy is crucial for reducing the complication risk [5,29]. A gradual dilation approach is recommended, primarily using undersized balloons to minimize vascular injury [5]. Also, it is recommended to focus on rings and webs in the first BPA procedures, leaving occluded vessels for later sessions. Continuous monitoring during and after BPA allows for the early identification and management of complications, ensuring they do not adversely affect long-term outcomes [15]. Finally, future randomized controlled trials are necessary to further define the role of PH-targeted therapy prior to BPA. The ongoing IMPACT-CTEPH trial (ClinicalTrials.gov Identifier NCT04780932) aims to provide insights into the effectiveness of PH-targeted monotherapy versus dual therapy before BPA. This trial holds promise in advancing our understanding and improving the management of patients undergoing BPA.

## 5. Limitations

The present analysis must be considered within the context of several limitations. First, the classification of peri-procedural complications relied on clinical experiences rather than advanced imaging modalities, potentially resulting in some misclassifications. Similarly, the complication severity was established based on descriptive measures rather than objective tools. Additionally, due to the retrospective nature of the study, we could not include all factors influencing the complication risk (e.g., lesion location, BPA interventionist experience). Furthermore, at our center, most patients receive dual PH-targeted medical therapy, especially patients with severe pulmonary hemodynamics. Since severe pulmonary hemodynamics may be associated with an increased risk of peri-procedural complications, no comparison could be made between the complication rate in patients who did and who did not receive PH-targeted medical therapy. Therefore, a direct relation between adequate PH-targeted medical therapy and a reduction in the complication risk cannot be assured from our data. Finally, the single-center character of this analysis must be underscored when generalizing the findings to other settings, particularly considering that PH-targeted therapy was used by most patients prior to BPA.

## 6. Conclusions

This is the first observational study to classify peri-procedural complications according to the ESC consensus statement on BPA treatments. The overall thoracic complication rate is 9%, with a significantly higher rate of up to 17% in the presence of severe pulmonary hemodynamics. Despite the retrospective and single-center nature of our study, our analysis establishes a benchmark for future studies on BPA-related complications. The association between increased pulmonary hemodynamics and (thoracic) complications underscores the necessity to investigate the role of PH-targeted therapy preceding BPA further.

## Figures and Tables

**Table 1:** Baseline characteristics of patient population.

		*n* (N = 87)	
Demographic information	Sex (female)	87	55 (63.2%)
	Age at diagnosis (years)	87	61.1 ± 14.0
	BMI > 30 kg/m^2^	69	22 (31.9%)
Medical history	Previous PEA	87	4 (4.6%)
	Systemic hypertension	85	29 (34.1%)
	Diabetes mellitus	86	8 (9.3%)
	CAD	85	3 (3.5%)
Clinical parameters	WHO FC III/IV	87	52 (59.8%)
	6MWD (m)	74	380 ± 142
	NTproBNP (pg/mL) *	81	344 [108–1788]
Pulmonary hemodynamics	mRAP (mmHg)	79	8.8 ± 5.6
	mPAP (mmHg)	86	38.9 ± 10.0
	PAWP (mmHg)	83	10.7 ± 5.0
	CO (L/Min)	82	5.2 ± 1.9
	PVR (WU)	81	6.4 ± 3.6
Anticoagulation type	DOAC	87	24 (27.6%)
	VKA		63 (72.4%)
PH-targeted medical therapy after the MDT	No	87	7 (8.0%)
	Mono		25 (28.7%)
	Dual		54 (62.1%)
	Triple		1 (1.1%)
	ERA	87	65 (74.7%)
	Riociguat	87	39 (44.8%)
	PDE5i	87	31 (35.6%)
	Oral prostanoid	87	1 (1.1%)

BMI = Body Mass Index; BPA = Balloon Pulmonary Angioplasty; CAD = Coronary Artery Disease; CO = Cardiac Output; DOAC = Direct Oral Anticoagulant; ERA = Endothelin Receptor Antagonist; IQR = Interquartile Range; MDT = multidisciplinary team; mPAP = mean Pulmonary Arterial Pressure; mRAP = mean Right Atrial Pressure; *n* = non-missing; NTproBNP = N-terminal Fragment of Pro-Brain Natriuretic Peptide; PEA = Pulmonary Endarterectomy; PAWP = Pulmonary Arterial Wedge Pressure; PDE5i = Phosphodiesterase-5 Inhibitor; PH = Pulmonary Hypertension; PVR = Pulmonary Vascular Resistance; WHO FC = World Health Organization Functional Class; VKA = Vita-min K Antagonist; 6MWD = 6-min walking distance Note: Data are given as mean ± standard deviation or n (%) or * median [IQR].

**Table 2:** Clinical characteristics during follow-up.

		At Baseline	At First BPA	At Last BPA
Clinical characteristics	WHO FC III/IV	52 (59.8%)	47 (54.0%)	15 (17.2%)
	6MWD (m)	380 ± 142	396 ± 122	442 ± 126
	Log NTproBNP (pg/mL)	5.9 ± 1.8	5.6 ± 1.5	5.1 ± 1.2
Pulmonary hemodynamics	mRAP (mmHg)	8.8 ± 5.6	7.1 ± 4.7	7.6 ± 4.1
	mPAP (mmHg)	38.9 ± 10.0	33.1 ± 11.0	29.2 ± 9.6
	PAWP (mmHg)	10.7 ± 5.0	11.8 ± 5.3	13.5 ± 5.8
	CO (L/Min)	5.2 ± 1.9	5.7 ± 1.6	6.0 ± 1.2
	PVR (WU)	6.4 ± 3.6	3.7 ± 2.3	2.7 ± 1.6

BPA = Balloon Pulmonary Angioplasty; CO = Cardiac Output; mPAP = Mean Pulmonary Arterial Pressure; mRAP = Mean Right Atrial Pressure; NTproBNP = N-terminal Fragment of Pro-Brain Natriuretic Peptide; PAWP = Pulmonary Arterial Wedge Pressure; PVR = Pulmonary Vascular Resistance; WHO FC = World Health Organization Functional Class; 6MWD = 6-Minute Walking Distance Note: Data are given as mean ± standard deviation or n (%).

**Table 3:** Overview of peri-procedural complications.

	All BPA Treatments (N = 87)	PVR ≤ 6.6 (N = 64)	PVR > 6.6 (N = 8)	*p*-Value ^1^	mPAP ≤ 45 (N = 69)	mPAP > 45 (N = 13)	*p*-Value ^1^
Total BPA count	426	296	53		317	85	
BPA count per patient	4.9 ± 1.6	4.6 ± 1.5	6.6 ± 1.5	0.002 **	4.6 ± 1.4	6.5 ± 1.7	<0.001 ***
Total number of complications	59 (13.8%)	33 (11.1%)	12 (22.6%)	0.002 **	40 (12.6%)	18 (21.2%)	0.007 **
Patients with complications ^2^	41 (47.1%)	26 (40.6%)	7 (87.5%)	0.020 *	30 (43.5%)	10 (76.9%)	0.027 *
Patients with thoracic complications ^2^	27 (31.0%)	17 (26.6%)	5 (62.5%)	0.051	19 (27.5%)	7 (53.8%)	0.10
All thoracic complications	38 (8.9%)	20 (6.8%)	9 (17.0%)	0.013 *	25 (7.9%)	12 (14.1%)	0.039 *
Hemoptysis	24 (5.6%)	10 (3.4%)	8 (15.1%)	<0.001 ***	15 (4.7%)	9 (10.6%)	0.026 *
*Mild*	19 (4.5%)	10 (3.4%)	4 (7.5%)	0.006 **	12 (3.8%)	7 (8.2%)	0.093
*Moderate*	5 (1.2%)	0 (0.0%)	4 (7.5%)	<0.001 ***	3 (0.9%)	2 (2.4%)	0.064
Vascular injury	8 (1.9%)	6 (2.0%)	0 (0.0%)	0.38	6 (1.9%)	1 (1.2%)	0.92
*Mild*	7 (1.6%)	5 (1.7%)	0 (0.0%)	0.43	5 (1.6%)	1 (1.2%)	0.97
*Moderate*	1 (0.2%)	1 (0.3%)	0 (0.0%)	0.76	1 (0.3%)	0 (0.0%)	0.69
Lung injury	4 (0.9%)	2 (0.7%)	1 (1.9%)	0.22	3 (0.9%)	1 (1.2%)	0.62
*Mild*	1 (0.2%)	1 (0.3%)	0 (0.0%)	0.76	1 (0.3%)	0 (0.0%)	0.69
*Moderate*	3 (0.7%)	1 (0.3%)	1 (1.9%)	0.083	2 (0.6%)	1 (1.2%)	0.41
Other thoracic complications	2 (0.5%)	2 (0.7%)	0 (0.0%)	>0.99	1 (0.3%)	1 (1.2%)	0.29
All non-thoracic complications	21 (4.9%)	13 (4.4%)	3 (5.7%)	0.48	15 (4.7%)	6 (7.1%)	0.23
Contrast allergy	9 (2.1%)	7 (2.4%)	0 (0.0%)	0.43	8 (2.5%)	1 (1.2%)	0.90
RHC complications	5 (1.2%)	3 (1.0%)	0 (0.0%)	0.55	4 (1.3%)	1 (1.2%)	0.81
Contrast nephropathy	1 (0.2%)	0 (0.0%)	0 (0.0%)		0 (0.0%)	1 (1.2%)	
Access site	0 (0.0%)	0 (0.0%)	0 (0.0%)		0 (0.0%)	0 (0.0%)	
Other complications	6 (1.4%)	3 (1.0%)	3 (5.7%)	0.031 *	3 (0.9%)	3 (3.5%)	0.12

BPA = Balloon Pulmonary Angioplasty; PVR = Pulmonary Vascular Resistance; RHC = Right Heart Catheterization. Note: Data are given as n (%) of total number of BPA treatments or as mean ± standard deviation. ^1^ * *p* < 0.05; ** *p* < 0.01; *** *p* < 0.001. ^2^ Three BPA treatments were associated with more than one complication.

## Data Availability

The data presented in this study are not publicly available due to privacy restrictions. Data may be shared upon request from the corresponding author, F.J. van Leusden.

## Supplementary Materials

The following supporting information can be downloaded at: https://www.mdpi.com/article/10.3390/jcm13154313/s1, Table S1: Clinical characteristics in subgroups of patients with PVR ≤ 6.6 > WU; Table S2: Clinical characteristics of subgroups of patients with mPAP ≤ 45 > mmHg; Table S3: Peri-procedural complications according to PVR status (including missing values for PVR); Table S4: Peri-procedural complications according to mPAP status (including missing values for mPAP).

## Abbreviations

BPA = Balloon Pulmonary Angioplasty; CTEPH = Chronic Thromboembolic Pulmonary Hypertension; ESC = European Society of Cardiology; IQR = Interquartile Range; mPAP = mean Pulmonary Arterial Pressure; PEA = Pulmonary Endarterectomy; PH = Pulmonary Hypertension; PVR = Pulmonary Vascular Resistance; RHC = Right Heart Catheterization; SD = Standard Deviation; WU = Woods Units; 6MWD = 6-minute walking distance; 95CI = 95% Confidence Interval.

## References

[1] Humbert M., Kovacs G., Hoeper M.M., Badagliacca R., Berger R.M.F., Brida M., Carlsen J., Coats A.J.S., Escribano-Subias P., Ferrari P. (2022). 2022 ESC/ERS Guidelines for the diagnosis and treatment of pulmonary hypertension. Eur. Respir. J..

[2] Moser K.M., Bioor C.M. (1993). Pulmonary Vascular Lesions Occurring in Patients with Chronic Major Vessel Thromboembolic Pulmonary Hypertension. Chest.

[3] Jenkins D. (2015). Pulmonary endarterectomy: The potentially curative treatment for patients with chronic thromboembolic pulmonary hypertension. Eur. Respir. Rev..

[4] Delcroix M., Lang I., Pepke-Zaba J., Jansa P., D’Armini A.M., Snijder R., Bresser P., Torbicki A., Mellemkjaer S., Lewczuk J. (2016). Long-term outcome of patients with chronic thromboembolic pulmonary hypertension. Circulation.

[5] Lang I.M., Andreassen A.K., Andersen A., Bouvaist H., Coghlan G., Escribano-Subias P., Jansa P., Kopec G., Kurzyna M., Matsubara H. (2023). Balloon pulmonary angioplasty for chronic thromboembolic pulmonary hypertension: A clinical consensus statement of the ESC working group on pulmonary circulation and right ventricular function. Eur. Heart J..

[6] Delcroix M., de Perrot M., Jaïs X., Jenkins D.P., Lang I.M., Matsubara H., Meijboom L.J., Quarck R., Simonneau G., Wiedenroth C.B. (2023). Chronic thromboembolic pulmonary hypertension: Realising the potential of multimodal management. Lancet Respir. Med..

[7] Sugimura K., Fukumoto Y., Satoh K., Nochioka K., Miura Y., Aoki T., Tatebe S., Miyamichi-Yamamoto S., Shimokawa H. (2012). Percutaneous Transluminal Pulmonary Angioplasty Markedly Improves Pulmonary Hemodynamics and Long-Term Prognosis in Patients with Chronic Thromboembolic Pulmonary Hypertension. Circ. J..

[8] Taniguchi Y., Miyagawa K., Nakayama K., Kinutani H., Shinke T., Okada K., Okita Y., Hirata K., Emoto N. (2014). Balloon pulmonary angioplasty: An additional treatment option to improve the prognosis of patients with chronic thromboembolic pulmonary hypertension. EuroIntervention.

[9] Sato H., Ota H., Sugimura K., Aoki T., Tatebe S., Miura M., Yamamoto S., Yaoita N., Suzuki H., Satoh K. (2016). Balloon Pulmonary Angioplasty Improves Biventricular Functions and Pulmonary Flow in Chronic Thromboembolic Pulmonary Hypertension. Circ. J..

[10] Lang I., Meyer B.C., Ogo T., Matsubara H., Kurzyna M., Ghofrani H.-A., Mayer E., Brenot P. (2017). Balloon pulmonary angioplasty in chronic thromboembolic pulmonary hypertension. Eur. Respir. Rev..

[11] Feinstein J.A., Goldhaber S.Z., Lock J.E., Ferndandes S.M., Landzberg M.J. (2001). Balloon Pulmonary Angioplasty for Treatment of Chronic Thromboembolic Pulmonary Hypertension. Circulation.

[12] Mizoguchi H., Ogawa A., Munemasa M., Mikouchi H., Ito H., Matsubara H. (2012). Refined Balloon Pulmonary Angioplasty for Inoperable Patients with Chronic Thromboembolic Pulmonary Hypertension. Circ. Cardiovasc. Interv..

[13] Kennedy M.K., Kennedy S.A., Tan K.T., de Perrot M., Bassett P., McInnis M.C., Thenganatt J., Donahoe L., Granton J., Mafeld S. (2022). Balloon Pulmonary Angioplasty for Chronic Thromboembolic Pulmonary Hypertension: A Systematic Review and Meta-analysis. CardioVascular Interv. Radiol..

[14] Ito R., Yamashita J., Ikeda S., Nakajima Y., Kasahara T., Sasaki Y., Suzuki S., Takahashi L., Komatsu I., Murata N. (2023). Predictors of procedural complications in balloon pulmonary angioplasty for chronic thromboembolic pulmonary hypertension. J. Cardiol..

[15] Wiedenroth C.B., Deissner H., Adameit M.S.D., Kriechbaum S.D., Ghofrani H.-A., Breithecker A., Haas M., Roller F., Rolf A., Hamm C.W. (2022). Complications of balloon pulmonary angioplasty for inoperable chronic thromboembolic pulmonary hypertension: Impact on the outcome. J. Heart Lung Transplant..

[16] Jaïs X., Brenot P., Bouvaist H., Jevnikar M., Canuet M., Chabanne C., Chaouat A., Cottin V., De Groote P., Favrolt N. (2022). Balloon pulmonary angioplasty versus riociguat for the treatment of inoperable chronic thromboembolic pulmonary hypertension (RACE): A multicentre, phase 3, open-label, randomised controlled trial and ancillary follow-up study. Lancet Respir. Med..

[17] Harris P.A., Taylor R., Thielke R., Payne J., Gonzalez N., Conde J.G. (2009). Research electronic data capture (REDCap)a metadata-driven methodology and workflow process for providing translational research informatics support. J. Biomed. Inform..

[18] Harris P.A., Taylor R., Minor B.L., Elliott V., Fernandez M., O’Neal L., McLeod L., Delacqua G., Delacqua F., Kirby J. (2019). The REDCap consortium: Building an international community of software platform partners. J. Biomed. Inform..

[19] van Thor M.C.J., Lely R.J., Braams N.J., Klooster L.T., Beijk M.A.M., Heijmen R.H., van den Heuvel D.A.F., Rensing B.J.W.M., Snijder R.J., Vonk Noordegraaf A. (2019). Safety and efficacy of balloon pulmonary angioplasty in chronic thromboembolic pulmonary hypertension in the Netherlands. Neth. Heart J..

[20] van Thor M.C.J., Snijder R.J., Kelder J.C., Mager J.J., Post M.C. (2020). Does combination therapy work in chronic thromboembolic pulmonary hypertension?. Int. J. Cardiol. Heart Vasc..

[21] Posit Team (2023). RStudio: Integrated Development Environment for R.

[22] Wickham H., Averick M., Bryan J., Chang W., McGowan L., François R., Grolemund G., Hayes A., Henry L., Hester J. (2019). Welcome to the tidyverse. J. Open Source Softw..

[23] Sjoberg Daniel D., Whiting K., Curry M., Lavery Jessica A., Larmarange J. (2021). Reproducible Summary Tables with the gtsummary Package. R J..

[24] Kawakami T., Matsubara H., Shinke T., Abe K., Kohsaka S., Hosokawa K., Taniguchi Y., Shimokawahara H., Yamada Y., Kataoka M. (2022). Balloon pulmonary angioplasty versus riociguat in inoperable chronic thromboembolic pulmonary hypertension (MR BPA): An open-label, randomised controlled trial. Lancet Respir. Med..

[25] Andersen A., Hansen J.V., Dragsbaek S.J., Maeng M., Andersen M.J., Andersen G., Mellemjkaer S., Ilkjær L.B., Nielsen-Kudsk J.E. (2022). Balloon pulmonary angioplasty for patients with chronic thromboembolic pulmonary hypertension previously operated by pulmonary endarterectomy. Pulm. Circ..

[26] Brenot P., Jaïs X., Taniguchi Y., Garcia Alonso C., Gerardin B., Mussot S., Mercier O., Fabre D., Parent F., Jevnikar M. (2019). French experience of balloon pulmonary angioplasty for chronic thromboembolic pulmonary hypertension. Eur. Respir. J..

[27] Ito R., Yamashita J., Sasaki Y., Ikeda S., Suzuki S., Murata N., Ogino H., Chikamori T. (2021). Efficacy and safety of balloon pulmonary angioplasty for residual pulmonary hypertension after pulmonary endarterectomy. Int. J. Cardiol..

[28] Chausheva S., Naito A., Ogawa A., Seidl V., Winter M.-P., Sharma S., Sadushi-Kolici R., Campean I.-A., Taghavi S., Moser B. (2019). Chronic thromboembolic pulmonary hypertension in Austria and Japan. J. Thorac. Cardiovasc. Surg..

[29] Lang I. (2023). Balloon Pulmonary Angioplasty for Chronic Thromboembolic Pulmonary Hypertension: Clinical Outcomes. Eur. Cardiol. Rev..

