# Supplementary material for: Complications of Balloon Pulmonary Angioplasty: A Comprehensive Analysis Based on the Latest ESC Consensus Statement
Source: J Clin Med. 2024 Jul 24;13(15):4313. doi: 10.3390/jcm13154313 (PMC11313613; doi:10.3390/jcm13154313)
Supplement: Supplementary file 1 [file jcm-13-04313-s001.zip › jcm-3044948-supplementary.pdf]

Supplementary Materials

Supplementary table 1: Clinical characteristics in subgroups of patients with PVR ≤ 6.6 > WU

		**PVR ≤6.6 (N=64)**	**PVR >6.6 (N=8)**
**Demographic information**	Sex (female)	40 (62.5%)	7 (87.5%)
	Age at diagnosis (years)	61.0 ± 14.3	66.5 ± 11.2
	BMI >30 kg/m^2	17 (37.0%)	0 (0.0%)
**Medical history**	Previous PEA	3 (4.7%)	0 (0.0%)
	Systemic hypertension	23 (36.5%)	3 (37.5%)
	Diabetes mellitus	5 (7.8%)	1 (12.5%)
	CAD	2 (3.2%)	1 (12.5%)
**Clinical parameters**	WHO FC III/IV	35 (60.3%)	5 (62.5%)
	6MWD (m)	403 ± 132	380 ± 50
	NTproBNP (pg/ml), median [IQR]	168 [99-344]	2,094 [1,338-2,527]
**Pulmonary hemodynamics**	mRAP (mmHg)	6.6 ± 4.1	6.5 ± 3.2
	mPAP (mmHg)	29.7 ± 8.7	46.9 ± 7.6
	PAWP (mmHg)	12.0 ± 5.4	9.1 ± 3.4
	CO (L/Min)	6.0 ± 1.6	4.5 ± 0.9
**BPA**	mean BPA count	4.6 ± 1.5	6.6 ± 1.5

BMI = Body Mass Index; BPA = Balloon Pulmonary Angioplasty; CAD = Coronary Artery Disease; CO = Cardiac Output; DOAC = Direct Oral Anticoagulant; IQR = Interquartile Range; mPAP = mean Pulmonary Arterial Pressure; mRAP = mean Right Atrial Pressure; NTproBNP = N-terminal Fragment of Pro-Brain Natriuretic Peptide; PEA = Pulmonary Endarterectomy; PAWP = Pulmonary Arterial Wedge Pressure; PH = Pulmonary Hypertension; PVR = Pulmonary Vascular Resistance; WHO FC = World Health Organization Functional Class; 6MWD = 6-minute walking distance Note: Data are given as mean ± standard deviation or n (%)

Supplementary table 2. Clinical characteristics of subgroups of patients with mPAP ≤ 45 > mmHg

		**mPAP ≤45 (N=69)**	**mPAP >45 (N=13)**
**Demographic information**	Sex (female)	44 (63.8%)	7 (53.8%)
	Age at diagnosis (years)	61.7 ± 13.5	58.6 ± 16.8
	BMI >30 kg/m^2	18 (35.3%)	3 (23.1%)
**Medical history**	Previous PEA	3 (4.3%)	1 (7.7%)
	Systemic hypertension	23 (33.8%)	5 (41.7%)
	Diabetes mellitus	8 (11.6%)	0 (0.0%)
	CAD	2 (2.9%)	1 (8.3%)
**Clinical parameters**	WHO FC III/IV	39 (62.9%)	8 (61.5%)
	6MWD (m)	397 ± 133	380 ± 59
	NTproBNP (pg/ml), median [IQR]	168 [99-344]	2,354 [880-2,450]
**Pulmonary hemodynamics**	mRAP (mmHg)	6.4 ± 4.0	10.2 ± 6.3
	PAWP (mmHg)	11.8 ± 5.3	11.6 ± 5.2
	CO (L/Min)	5.8 ± 1.7	5.1 ± 1.0
	PVR (WU)	3.2 ± 1.6	8.1 ± 2.3
**BPA**	mean BPA count	4.6 ± 1.4	6.5 ± 1.7

BMI = Body Mass Index; BPA = Balloon Pulmonary Angioplasty; CAD = Coronary Artery Disease; CO = Cardiac Output; DOAC = Direct Oral Anticoagulant; IQR = Interquartile Range; mPAP = mean Pulmonary Arterial Pressure; mRAP = mean Right Atrial Pressure; NTproBNP = N-terminal Fragment of Pro-Brain Natriuretic Peptide; PEA = Pulmonary Endarterectomy; PAWP = Pulmonary Arterial Wedge Pressure; PH = Pulmonary Hypertension; PVR = Pulmonary Vascular Resistance; WHO FC = World Health Organization Functional Class; 6MWD = 6-minute walking distance Note: Data are given as mean ± standard deviation or n (%)

Supplementary table 3. Peri-procedural complications according to PVR status (including missing values for PVR)

	**PVR ≤6.6 (N=64)**	**PVR >6.6 (N=8)**	**PVR missing(N=15)**	**p-value[1]**
**Total BPA count**	296	53	77	
**Mean BPA count**	4.6 ± 1.5	6.6 ± 1.5	5.1 ± 1.6	0.006**
**Total number of complications[2]**	33 (11.1%)	12 (22.6%)	14 (18.2%)	0.008**
**Patients with complications[3]**	26 (40.6%)	7 (87.5%)	8 (53.3%)	0.031*
**Patients with thoracic complications[3]**	17 (26.6%)	5 (62.5%)	5 (33.3%)	0.14
**Thoracic complications**	20 (6.8%)	9 (17.0%)	9 (11.7%)	0.048*
Hemoptysis during the procedure	10 (3.4%)	8 (15.1%)	6 (7.8%)	0.001**
Mild	10 (3.4%)	4 (7.5%)	5 (6.5%)	0.024*
Moderate	0 (0.0%)	4 (7.5%)	1 (1.3%)	<0.001***
Vascular injury	6 (2.0%)	0 (0.0%)	2 (2.6%)	0.57
Mild	5 (1.7%)	0 (0.0%)	2 (2.6%)	0.53
Moderate	1 (0.3%)	0 (0.0%)	0 (0.0%)	0.84
Lung injury	2 (0.7%)	1 (1.9%)	1 (1.3%)	0.45
Mild	1 (0.3%)	0 (0.0%)	0 (0.0%)	0.84
Moderate	1 (0.3%)	1 (1.9%)	1 (1.3%)	0.21
Other thoracic complications	2 (0.7%)	0 (0.0%)	0 (0.0%)	>0.99
**Non-thoracic complications**	13 (4.4%)	3 (5.7%)	5 (6.5%)	0.54
Contrast allergy	7 (2.4%)	0 (0.0%)	2 (2.6%)	0.55
Complications associated with RHC	3 (1.0%)	0 (0.0%)	2 (2.6%)	0.33
Contrast nephropathy	0 (0.0%)	0 (0.0%)	1 (1.3%)	
Access site	0 (0.0%)	0 (0.0%)	0 (0.0%)	
Others	3 (1.0%)	3 (5.7%)	0 (0.0%)	0.035*

Abbreviations: BPA = Balloon Pulmonary Angioplasty; PVR = Pulmonary Vascular Resistance; RHC = Right Heart Catheterization Note: Data are given by mean ± standard deviation or as n (%) of number of BPA treatments

[1]p <0.05; ** p <0.01; *** p <0.001

[2]Three BPA treatments were associated with more than one complication

[3]n (%) of total number of patients

Supplementary table 4. Peri-procedural complications according to mPAP status (including missing values for mPAP)

	mPAP ≤45 (N=69)	mPAP >45 (N=13)	mPAP missing (N=5)	p-value[1]
**Total BPA count**	317	85	24	
**Mean BPA count**	4.6 ± 1.4	6.5 ± 1.7	4.8 ± 0.8	0.003**
**Total number of complications**[2]	40 (12.6%)	18 (21.2%)	1 (4.2%)	0.011*
**Patients with complications**[3]	30 (43.5%)	10 (76.9%)	1 (20.0%)	0.040*
**Patients with thoracic complications**[3]	19 (27.5%)	7 (53.8%)	1 (20.0%)	0.15
**Thoracic complications**	25 (7.9%)	12 (14.1%)	1 (4.2%)	0.094
Hemoptysis during the procedure	15 (4.7%)	9 (10.6%)	0 (0.0%)	0.045*
Mild	12 (3.8%)	7 (8.2%)	0 (0.0%)	0.14
Moderate	3 (0.9%)	2 (2.4%)	0 (0.0%)	0.14
Vascular injury	6 (1.9%)	1 (1.2%)	4 (4.2%)	0.69
Mild	5 (1.6%)	1 (1.2%)	4 (4.2%)	0.60
Moderate	1 (0.3%)	0 (0.0%)	0 (0.0%)	0.88
Lung injury	3 (0.9%)	1 (1.2%)	0 (0.0%)	0.77
Mild	1 (0.3%)	0 (0.0%)	0 (0.0%)	0.88
Moderate	2 (0.7%)	1 (1.2%)	0 (0.0%)	0.63
Other thoracic complications	1 (0.3%)	1 (1.2%)	0 (0.0%)	0.37
**Non-thoracic complications**	15 (4.7%)	6 (7.1%)	0 (0.0%)	0.26
Contrast allergy	8 (2.5%)	1 (1.2%)	0 (0.0%)	0.79
Complications associated with RHC	4 (1.2%)	1 (1.2%)	0 (0.0%)	0.82
Contrast nephropathy	0 (0.0%)	1 (1.2%)	0 (0.0%)	
Access site	0 (0.0%)	0 (0.0%)	0 (0.0%)	
Others	3 (0.9%)	3 (3.5%)	0 (0.0%)	0.24

Abbreviations: BPA = Balloon Pulmonary Angioplasty; PVR = Pulmonary Vascular Resistance; RHC = Right Heart Catheterization Note: Data are given by mean ± standard deviation or as n (%) of number of BPA treatments

[1]p <0.05; ** p <0.01; *** p <0.001

[2]Three BPA treatments were associated with more than one complication

[3]n (%) of total number of patients
